# Comparison of Patient Adverse Drug Reaction Reporting Systems in Nine Selected Countries

**DOI:** 10.3390/ijerph19084447

**Published:** 2022-04-07

**Authors:** Wiwan Worakunphanich, Sitaporn Youngkong, Wimon Suwankesawong, Claire Anderson, Montarat Thavorncharoensap

**Affiliations:** 1 Doctor of Philosophy Program in Social, Economic, and Administrative Pharmacy, Department of Pharmacy, Faculty of Pharmacy, Mahidol University, Bangkok 10400, Thailand; toon_punch@hotmail.com; 2 Thai Traditional Medicine Research Institute, Department of Thai Traditional and Alternative Medicine, Ministry of Public Health, Nonthaburi 11000, Thailand; 3 Health Technology Assessment Graduate Program, Mahidol University, Bangkok 10400, Thailand; sitaporn.you@mahidol.edu; 4 Social and Administrative Pharmacy Excellence Research (SAPER) Unit, Department of Pharmacy, Faculty of Pharmacy, Mahidol University, Bangkok 10400, Thailand; 5 The College of Pharmaceutical and Health Consumer Protection of Thailand, Bangkok 10330, Thailand; wmswyala@gmail.com; 6 School of Pharmacy, University of Nottingham, Nottingham NG7 2RD, UK; claire.anderson@nottingham.ac.uk

**Keywords:** patient adverse drug reaction reporting, patient reporting system, ADR reporting, pharmacovigilance

## Abstract

Patients are recognized as important players in the pharmacovigilance system. This study aims to describe and compare the characteristics of patient reporting systems, reporting forms, awareness raising-activities, and the statistics related to patient reporting in the selected countries. Fifteen countries (eight Western countries and seven Asian countries) were purposively selected. A questionnaire survey was distributed to national pharmacovigilance authorities in those countries. Nine countries (five Western countries and four Asian countries) returned the questionnaire. A review of the websites of national pharmacovigilance centres was conducted. The proportion of patient reports in the selected Western countries ranged from 57.83% to 14.37%, while it was accounted for less than 1% in the selected Asian countries. Currently, patients in all nine countries can report adverse drug reactions online via a website. The number of clicks from the national pharmacovigilance website to reach the online reporting form range from one to five clicks. Countries with higher patient reporting rates seemed to share the following characteristics; provision of feedback, engagement with patient organizations, and implementation of several activities to raise the awareness of the general public on the importance of pharmacovigilance. To increase the number of patient reports, the strengths of each country’s system should be adopted.

## 1. Introduction

Spontaneous reporting systems are the most commonly used method in pharmacovigilance. The main function of Spontaneous adverse drug reaction (ADR) reporting systems is the early identification of signals of new, rare, and serious ADRs [1]. The WHO explained patient reporting as reporting of ADR by the general public [1]. Patient report is described as a way to increase the number of reports in order to enable earlier detection of ADRs [2]. Until recently, spontaneous reporting systems have relied exclusively on healthcare professionals (HCPs). Before the implementation of European Union (EU) pharmacovigilance legislation in 2012 [3], which allows and mandates member states to encourage patients to report suspected adverse drug reactions directly to the regulatory agency, very few countries have initiated a patient reporting system.

To date, patients are recognized as an integral part of the pharmacovigilance system [2]. Compared to HCP reports, in which ADRs were reported by HCPs, patient reports contained a higher median number of suspected ADRs per report, with more detailed descriptions [4]. It is quite clear that patient reports add useful information, especially on experiences and impacts of ADRs on daily life [5] and quality of life [6]. Evidence also indicates that new and novel ADRs can be detected through patient reports [1] and that combining patient and HCP reports generated more potential signals than HCP reports alone [4]. Nevertheless, the number of patient reports is still limited [5].

Characteristics of the patient reporting system are one of the main factors contributing to the number of patient reports. To date, most countries have set up a system for patient reporting of ADRs. According to the WHO handbook [1], ADR reporting forms should be easily accessed on the agencies’ websites. The method to report and the reporting form should also be simple and easy to understand by a layperson. According to a previous review [7], the characteristics of patient reporting systems are diverse. Each country has been using its own patient reporting system and patient reporting form. The number of patient ADR reports in each country also varies [7].

Existing reviews examining characteristics of patient reporting systems were published in 2012 and 2014, with limited information from Asian countries [2,7]. Due to the potential change in the patient reporting system and the absence of some aspects of patient reporting systems, a new review is warranted. Our study aims to describe and compare characteristics of patient reporting systems as well as patient reporting forms, the number of patient reports, and activities to promote patient reporting systems in the selected countries with high patient report rates and the selected Asian countries. This study will help identify the strengths and key strategies of each national system to promote patient reporting systems that could give important insights for application in other settings.

## 2. Materials and Methods

### 2.1. Country Selection

Fifteen countries were purposively selected for the study. First, the eight countries with patient reporting rates of more than 10% of the total report based on the previous review [7] were purposively selected (i.e., Belgium, Canada, Denmark, The Netherlands, Morocco, Sweden, the United Kingdom (UK), and the United States of America (USA)). We then purposively selected the following 7 Asian countries that have patient reporting ADR systems; India, Japan, Malaysia, the Philippines, South Korea, Taiwan, and Thailand.

### 2.2. Study Design

#### 2.2.1. A Cross-Sectional Survey

Based on the literature review, a questionnaire was developed. The content validity of the questionnaire was evaluated by 4 experts to ensure that it covered all key information relevant to the objectives of the study. The expert panel also rechecked the appropriateness of written language and the order of the questions. The final version of questionnaire consisted of 5 parts as follows: (1) Characteristics of pharmacovigilance system (i.e., year of commencement of the spontaneous report system, year of joining the WHO Programme for International Drug Monitoring, number of full-time staff at pharmacovigilance centres, and types reported); (2) Characteristics of the patient reporting system (i.e., year of commencement of the patient reporting system, channel to submit the report, and type of products that patient can report); (3) Characteristics of patients reporting form (i.e., characteristics of HCPs and patients reporting form, characteristics of the form used to report AEs associated with western and herbal medicine, item in the patient reporting form, and method to access a patient reporting from); (4) Statistics regarding the numbers of patient and HCP report during 2015–2019; and (5) Supportive activities related to patient report (i.e., feedback to reporters, acknowledge of the receipt of ADR reports, and methods for promoting patient reporting systems).

A questionnaire survey along with an invitation was sent to the national pharmacovigilance authorities of the 15 selected countries via e-mail from February to September 2020. E-mail addresses were identified from the website of the national pharmacovigilance. The invitation included a cover letter explaining the purpose and method of the survey and a self-administered questionnaire. Reminders were then sent by e-mail after 4 weeks to the non-response samples by using the same contact mode as the first invitation.

#### 2.2.2. Review of the National Pharmacovigilance Centres’ Website

The websites of national pharmacovigilance centres [8,9,10,11,12,13,14,15,16] were explored to identify the following information; characteristics of patient and HCP reporting forms, channels to submit a report, the method to access the reporting form, the total number of fields in the reporting forms, the total number of mandatory fields in the reporting form, the number of clicks to reach online report and to download paper reporting form, availability of specific features (i.e., dropdown list, free text description, short VDO, help menu, or ability to upload pictures), and activities/campaigns that promote patient reporting systems to the general public).

If the pre-specified data were missing from the questionnaire survey and/or website of the national pharmacovigilance centres, that information was searched for via Pubmed and Google scholar.

The study was conducted after final approval from the Institutional Review Board of Mahidol University, Faculty of Dentistry/Faculty of Pharmacy (COA.No.MU-DT/PY-IRB 2020/010.2701).

#### 2.2.3. Data Analysis

Percentages of patient reports to the total reports were calculated as the total number of patient reports of ADRs in 5 years (2015–2019) divided by the total number of ADRs reports in 5 years (2015–2019) and multiplied by 100. The number of patient reports of ADRs per year per million inhabitants was calculated as the average annual number of patient ADR reports during 2015–2019 divided by the average number of the population during 2015–2019 [17]. The number of total ADR reports in 2019 per full-time staff was also calculated. The total number of fields and mandatory fields in the reporting forms, the required information in the reporting forms, and the number of clicks to reach the reporting form were all counted via the website of the national pharmacovigilance centres. Then, the comparison between paper and online forms was made (i.e., the number of fields and mandatory fields). In addition, the differences between the reporting forms for Western and herbal medicine were explored.

## 3. Results

Of the 15 questionnaires distributed, nine were returned, resulting in a response rate of 60%. Of the total respondents, there were five (55.56%) countries with high patient reporting rates (i.e., Belgium, Canada, Denmark, the Netherlands, and the United Kingdom (UK)) and four (44.44%) Asian countries (i.e., Malaysia, the Philippines, Taiwan, and Thailand).

### 3.1. Statistics Regarding the Number of ADRs Report

Statistics regarding the number of ADRs reports were retrieved from the survey and are shown in Table 1. In terms of the average number of patient reports during the last 5 years (2015–2019), Canada ranked the highest (50,091 reports). Nevertheless, it should be noted that the number of patients reports in Canada shown above did not include only reports from patients but also from lawyers and other non-HCPs. With respect to the share of patient reports to the total reports, the highest patient reports accounted for 57.83% of the total reports in the Netherlands. The percentage of patient reports in the four selected Asian countries was quite low, ranging from 0.03% in the Philippines to 0.13% of the total reports in Malaysia. In terms of the number of patient reports per inhabitant per year, the highest was found in Denmark (467 per million inhabitants). In contrast, the number of patient reports in the selected Asian countries ranged only from 0.01 in the Philippines to 0.86 per million inhabitants in Malaysia. The number of full-time staff at the pharmacovigilance centre varied from three (the Philippines) to 130 staff (the UK), see Table 1. In terms of the number of total ADRs reports per full-time staff in 2019, the highest ratio was observed in Thailand (2971 reports per one staff).

### 3.2. Characteristics of the Spontaneous Reporting Systems and Patient Reporting Systems

All countries have initiated spontaneous reporting systems since the 20th century (Table 2). The first country which implemented a patient reporting system was Canada in 1965. Denmark is the first EU Member State to introduce direct patient reports since 2003. Among the selected Asian countries, Taiwan was the first country to establish patient reporting systems. In all countries except Canada and Taiwan, patient reporting systems were launched after the year 2003 (Table 2).

### 3.3. Characteristics of Channel and Form of Patient Reporting

In all countries, patients can report via postal mail, online via a website, and telephone. In the following countries: Belgium, Denmark, and the UK, patients can now report using a mobile phone app (Table 3). The number of clicks from the national pharmacovigilance website to reach the online reporting form ranges from one to five. The number of clicks from the website to download the paper reporting forms ranged from two to six (Table 2).

### 3.4. Characteristics of Patient Reporting Forms

In all countries, the same patient reporting form was used for both herbal medicine and Western medicine. Nevertheless, ADR reports related to herbal medicines in Taiwan are sent to different organizations (Table 3). The paper and online reporting forms for patients are different in all nine countries except Malaysia and Taiwan (Table 3). With respect to access to the reporting form, patients in all nine countries can find the reporting forms via a website (Table 4). Furthermore, patients in the UK and Malaysia can also find the reporting form at a hospital and in primary care.

In the following countries, the paper forms to be completed by HCPs and patients are different: Belgium, Malaysia, the Netherlands, and the UK. The total number of fields in the patient paper form ranged from 21 to 39, see Table 5. The number of mandatory fields in the paper form for patients ranged from 0 to 20. For the paper form, the common mandatory fields were the age or the birth date of the patient, suspected medicine, symptoms of ADR, and contact information of the reporter (Table 6). The free-text fields were used for reporting the suspected medicine and symptoms of ADR in all countries. For online reports, the forms for HCPs and patients were different in Belgium, Denmark, Malaysia, Thailand, and the UK. The total number of fields in the patient online form ranged from 18 to 45 (Table 5). The number of mandatory fields for patients in the online form for patients ranged from 0 (i.e., Taiwan) to 20 (i.e., the UK), see Table 4. For patient online reporting forms, the name or initials of patients, suspected medicine, and symptoms of ADR were required in all countries except Taiwan (Table 7). The contact information of the reporter (e.g., e-mail, telephone number, and address) were required in all countries except Belgium and Taiwan. Drop-down lists were presented when reporting the name of a suspected medicine in all countries except Canada. Free-text fields were provided to allow patients to describe symptoms in all countries except the UK, where a drop-down list is provided. In the Netherlands, patients can also upload a picture (e.g., suspected side effects or a discharge letter from a hospital) to the online form. Similarly, medical records can be uploaded to the online form in Taiwan. In all countries, except Thailand and Malaysia, either help menus or explanations/examples were provided for each question in the online form to facilitate the completion of the reporting form.

### 3.5. Activities Regarding of Adverse Drug Reactions Reporting by Patient

In Belgium, Canada, the Netherlands, and the UK, feedback was given to the reporters (Table 8). In the UK and Canada, the link to the online database of ADR reports received by the pharmacovigilance centre were sent as feedback to the reporters. In the Netherlands, feedback will be provided in specific cases (e.g., when doctor advice is required, or when the patient asks a question). In all countries, the reporters were contacted for further information or if clarification was needed.

All countries except Thailand and Taiwan promoted their patient reporting systems via media (e.g., television, radio) and social media (e.g., Facebook, Twitter, or YouTube); see Table 8. In some countries (i.e., Denmark, Malaysia, the Netherlands, and the UK), patient reporting systems were also promoted through HCPs. The patient organizations are an important link between patients and national pharmacovigilance centres to promote patient reporting systems in Denmark, the Netherlands, and the UK. Some countries (i.e., Belgium, the Netherlands, and the UK) created activities to educate the general public on the importance of reporting.

## 4. Discussion

As expected, we could observe that the five selected Western countries have higher patient reporting rates than the four Asian countries. The number of patient reports per million inhabitants was relatively high in the five selected Western countries. Nevertheless, it should be noted that the number of reports as well as the report rate per inhabitant could not be directly compared with previous studies [6] due to the different definitions of patient reports used in the calculation.

Consistent with the previous study [19], the countries with high numbers of patient reporting seem to establish a national reporting system or patients reporting system earlier, and joined the WHO Programme for International Drug Monitoring (PIDM) earlier. As it is well known that lack of awareness is an important factor for under-reporting [20], we observed that the countries with a higher number of reports generally employed several strategies to promote awareness. In addition, countries with higher reporting rates seemed to intensively engage with consumer organizations to promote the awareness and acceptance of pharmacovigilance among the general public (i.e., Denmark, the Netherlands, and the UK). In the UK, the Medicines and Healthcare Products Regulatory Agency (MHRA) collaborated with patient organizations to produce ADR reporting guidelines for specific patient groups [21]. In the Netherlands, Lareb intensively engaged with consumer organizations to promote the awareness and acceptance of pharmacovigilance among the general public. Two out of 10 board members of Lareb are patient representatives [22]. In addition, countries with higher reporting rates seem to hold campaigns to raise awareness and promote ADR reporting among the public (i.e., Belgium, the Netherlands, and the UK). In the Netherlands, a joint campaign with the central bureau of drugstores was launched to promote the reporting of over-the-counter (OTC) drugs by distributing leaflets explaining how to report ADRs related to OTC drugs [22]. In the UK, the ‘Every Report Counts’ campaign was launched with the aim of increasing awareness, engagement, and improving understanding of the reporting scheme to the general public and HCPs [12]. In Belgium, a campaign, “2031 needs you—no new medicines without your help”, was launched to raise public awareness [9]. The lack of promotion activities among the four Asian countries might be due to the limited human resources. We observed that the workload in terms of the number of reports per staff was relatively high in Asian countries, as compared to the Western countries. To improve patient reporting systems, further staff may be required in such settings.

Another reason that could explain the higher report rate among the four European countries is probably the collaboration and harmonization across the EU countries. Unlike Asian countries, the European countries have issued Pharmacovigilance legislation, which requires all Member States to develop patient reporting systems and aims to empower patients through reporting and participation since 2012. In addition, the EU pharmacovigilance system is built on the principles of engagement and collaboration within the EU network.

In terms of channels of patient reporting, all countries offered several ways for patients to report ADRs. This is consistent with the WHO guidelines, which suggest that the means of reporting for the general public should be as easy and cheap as possible [1]. A previous review [7] found that the most common reporting channel is via a paper form coupled with an online form. With the advance in technology, our study found that patients in all nine countries can currently report ADRs online and via telephone. The benefits of online reporting are that it increases the number of reports [7], and it could also reduce staff workload as the reports can be automatically imported into databases, which saves time and reduces the number of staff required. However, alternative channels such as telephone reporting, or paper forms that can be returned by mail or fax should still be provided, especially for less developed countries where literacy rates or internet access is limited. In Canada and the UK, patients can submit the paper form using a postage-paid label, which could help in facilitating the report submission [11,12]. In many countries such as Belgium, Denmark, and the UK, patients can now report via a mobile phone app. It should be noted that an app was also available in the Netherlands in 2016 [23]. However, the app is currently unavailable for download. As previous studies found, there is a growing interest in mobile apps for reporting ADRs, as it is a faster and easier way to report and provides easier access to the reporting form [20,24,25]. Further studies on benefits and appropriate features associated with patient reporting via mobile apps should be explored. Although there is a positive attitude toward reporting ADRs via social media [25], patients in the nine selected countries cannot report ADRs via social media. Social media is a potential channel to report ADRs and promote patient reporting to the general public, especially in Asian countries, where time spent on social media is extensive [26]. Further study on the values and suitable features of online social media to report ADRs should be examined.

To facilitate reporting, ADR reporting forms should be easily accessed via several channels [1]. In terms of access to reporting form, the UK provides a good example as the reporting form can be accessed not only online but also at hospitals, community pharmacies, and in primary care. For the online reporting form, all nine countries provided the link to online reporting through the national pharmacovigilance centre’s website. To facilitate the reporting, an icon to complete the report should be easily navigated on the pharmacovigilance agency’s website. According to our study, the number of clicks to reach an online report and paper reporting form is not higher than six, indicating that access to the form is not difficult.

The reporting form was an important tool for improving ADRs reporting. A previous review found that difficulties with ADRs reporting forms are one of the important barriers for patients to report ADRs [27]. Consistent with the WHO guideline [1], patient and HCP forms could be different, in that the form for patients should be more simple and easier to complete by including layperson language and should also address some patient-specific questions. Although there are some international recommendations about mandatory fields in the patient reporting form [1,28], our study found that the mandatory fields in the patient reporting form varied across countries. Therefore, international guidelines on a standard patient reporting form should be promoted and emphasized. According to our review, there is no difference between the online form of HCPs and patients in Canada, the Netherlands, the Philippines, and Taiwan. However, in these countries, explanations, examples, or help menus were provided for each question. It should also be noted that for the countries where the literacy rate and education level are not always high, the form for patient reports should be different from the HCP report. For online reports, the number of mandatory cells should be kept minimal and a tradeoff considered between essential information and report rate.

When looking at the characteristics of the online form, patients in all nine countries can describe their symptoms using free text except in the UK, where a drop-down list is provided. Based on the previous study, the choices of symptoms provided in the menu should not be so complicated that the patients are unable to choose [29]. The strength in the Netherlands and Taiwan is that patients can upload pictures or files to the online form.

After submitting a report, patients should receive feedback or an overview of similar ADRs that have been reported [1]. Individualized feedback is particularly important for serious ADRs and when reporters ask specific questions [30]. Previous studies found that the provision of feedback was a motive for a patient to report ADRs [4,27] and that 61% of the reporters would have liked feedback [29]. Thus, the provision of feedback should become a mandatory part of the pharmacovigilance system. Nevertheless, according to our study, feedback seemed to be given in the countries with high report rates (i.e., Belgium, Canada, the Netherlands, and the UK). After submission, a reporter in Denmark receives an e-mail from the Danish Medicines Agency (DKMA) confirming the receipt with a reference number [22]. In the UK, reporters are encouraged to provide their full address or e-mail so that the MHRA can acknowledge receipt of the report and follow up for further information if necessary [12]. In the Netherlands, a receipt confirmation e-mail along with a report summary will be sent upon request [31]. Furthermore, individualized feedback will be provided in case of serious reports, in response to a specific question, to issue a recommendation to the reporter to visit a doctor, and in cases with legal implications [22]. In the Netherlands, the study found that patients who submitted a report from 2012 to 2013 were satisfied with feedback received from the pharmacovigilance centres either in terms of personalized feedback or an acknowledgement letter [32]. It should be noted that no feedback was provided by the selected Asian countries. This is probably due to the high workload of staff or the lack of clear policy in the pharmacovigilance centres.

Since the coronavirus disease (COVID-19) pandemic, six countries, including Belgium, Denmark, the Netherlands, the UK, the Philippines, and Taiwan, developed a specific channel in the national pharmacovigilance website for patients to report adverse events related to COVID-19 vaccines [8,9,10,13,14,33]. In Thailand, a specific mobile app for patients reporting ADRs from COVID-19 vaccine to Adverse Events Following Immunization (AEFI) system under the Department of Disease Control was developed. In the UK, patients are able to report suspected ADRs to medicines, vaccines, medical devices, and test kits used in COVID-19 treatment via the Coronavirus Yellow Card reporting site [33]. The most common information required in the report is the name of the COVID-19 vaccine, batch or lot number of the COVID-19 vaccine, and details of suspected adverse events.

In the past, it was found that the number of ADR reports increased sharply during a pandemic (for example, reporting of ADRs associated with injection of H1N1vax substantially increased during the H1N1 influenza pandemic) [34]. During the COVID-19 pandemic, many countries encouraged patients to report ADRs related to the COVID-19 vaccine and treatment. For example, the UK launched the COVID-19 campaign to encourage those receiving the COVID-19 vaccine to report a suspected side effect to the MHRA’s yellow card scheme on the Coronavirus yellow card reporting website [33]. Each country should take the opportunity of the COVID-19 pandemic to promote awareness of the importance and availability of patient reporting.

Although our study provides useful information on the unique features of patient reporting systems used in each country from which other settings can learn, it has some limitations. First, we relied on the response from a review of the national pharmacovigilance websites and questionnaire survey data. In addition, we could not be able to identify the position of the staff who responded to our survey. Nevertheless, we have evaluated the validity of the responses from the survey with the information provided by the National Pharmacovigilance offices whenever possible. We found that the responses from the survey seemed to be valid. As a purposive selection of countries was made and only five Western and four Asian countries responded, therefore, the generalizability of the findings to other Western and Asian countries should be made with caution.

## 5. Conclusions

Patient reporting systems in each of the selected countries are not too diverse. In all countries, patients can currently report ADRs online, by postal mail, or by telephone. However, there is still room for improvement in the selected Asian countries, especially in terms of the provision of patient feedback, engagement from the respective organization, and implementation of campaigns or activities to promote awareness and understanding of the patient reporting system. It would also be useful to adopt the strengths form each country. In addition, the patient reporting form should be designed to be simple and easily completed and include examples or explanations. The method to access the reporting form should also be easy. Moreover, there should be a sufficient number of staff in reporting centres to provide feedback and to enable promotion and awareness-raising activities.

## Figures and Tables

**Table 1:** Statistics regarding ADRs report.

Country	Total Number of ADRs Reports in 5 Years (2015–2019) ^a^	Total Number of Patient Reports of ADRs in 5 Years (2015–2019) ^a^	Total Number of HCP Reports of ADRs in 5 Years (2015–2019) ^a^	% of Patient Reports to the Total Reports	Number of Patient Reports of ADRs per Year per Million Inhabitants ^b^	Number of Full-Time Staff at Pharmacovigilance Centre ^a^	Number of Total ADR Reports in 2019 per Full Time Staff
Belgium	3780	1717	2053	45.42	30.07	31	30.23 (937/31)
Canada	348,580 ^c^	50,091 ^c^	184,345 ^c^	14.37	272.79	N/A	N/A
Denmark	35,247	13,374	21,873	37.94	466.70	22	296 (6512/22)
Netherlands	66,002	38,172	27,120	57.83	448.55	50	284.16 (14,208/50)
UK	210,938	37,182	103,851	17.63	111.47	130	336.74 (43,776/130)
Malaysia	99,493	134	89,070	0.13	0.86	25	1199.32 (29,983/25)
Philippines	22,720	7	5516	0.03	0.01	3	1487 (4461/3)
Taiwan	73,799	75	57,141	0.10	0.63	21	749.86 (15,747/21)
Thailand	224,756	205	216,149	0.09	0.59	13	2971.38 (38,628/13)

^a^: Data from survey, ^b^: calculated as the average annual number of patient ADR reports during 2015–2019/average number of population during 2015–2019. The number of population for each country was derived from United Nations [17]. ^c^: The number of patients report during 2015–2017 included reports from consumer, lawyers or other non-HCPs while the number in 2019 was from general population. The number of patients reports and HCPs reports in 2018 was calculated using the same proportion as reported in 2019. N/A: not available.

**Table 2:** Characteristics of the spontaneous report systems and patient reporting systems.

Country	Website ^a^	Year of Commencement of the ADR Reporting System ^b^	Year of Commencement of the Patient ADR Reporting System ^b^	Year of Joining WHO PIDM ^b^	Number of Clicks to Reach Online Report ^a^	Number of Clicks to Download Paper Reporting Form ^a^
Belgium	www.fagg-afmps.be	1976	2012	1977	3	3
Canada	www.hc-sc.gc.ca	1965	1965	1968	3	4
Denmark	www.laegemiddelstyrelsen.dk	1968	2003	1971	4	N/A
Netherlands	www.lareb.nl	1963 [18]	2003	1968	1	2
UK	www.gov.uk/mhra	1964	pilot in 2005	1968	3 ^c^	4
Malaysia	www.npra.gov.my	1987	pilot in 2007	1990	2	2
Philippines	www.fda.gov.ph	1994	pilot in 2008	1995	2	2
Taiwan	www.fda.gov.tw	1998	1998	N/A	5	6
Thailand	www.fda.moph.go.th	1983	2010	1984	2	3

^a^: Data from website of national pharmacovigilance centres, ^b^: Data from survey, ^c^: 1 click from yellow card website, WHO PIDM: WHO Programme for International Drug Monitoring, N/A: not available.

**Table 3:** Characteristics of channel and form of patient reporting.

Country	Channel of Patient Reporting ^a^						Different Form for Reporting Western and Herbal Medicine ^a^	Different Form for Paper and Online ^a^
	Postal Mail	E-Mail	Online	Fax	Tel	Mobile Apps		
Belgium	✓	✓	✓		✓	✓ ^b^	No	Yes
Canada	✓		✓	✓	✓		No	Yes
Denmark	✓ ^b^	✓ ^b^	✓		✓	✓ ^b^	No	N/A
Netherlands	✓		✓		✓		No	Yes
UK	✓	✓ ^b^	✓	✓	✓	✓	No	Yes
Malaysia	✓	✓	✓	✓	✓		No	No
Philippines	✓	✓	✓		✓		No	Yes
Taiwan	✓	✓	✓	✓	✓		No ^b,c^	No
Thailand	✓	✓	✓	✓	✓		No	Yes

^a^: Data from website of national pharmacovigilance centres, ^b^: Data from survey, ^c^: Herbal medicines AE reporting form has shared similar essential components with reporting form for pharmaceutical products. However, herbal medicines AE reporting system in Taiwan is not operated by Taiwan’s pharmacovigilance centre. N/A: not available.

**Table 4:** Characteristics of patient reporting form.

Country	Paper Form							Online Form		
	Different Form for HCPs and Patients ^a^	No. of Mandatory Fields for HCPs ^b^	No. of Mandatory Fields for Patients ^b^	Method to Access the Form ^a,b^				Different Form for HCPs and Patients ^a^	No. of Mandatory Fields for HCPs ^b^	No. of Mandatory Fields for Patients ^b^
				Hospital	Community Pharmacist	Primary Care	Website			
Belgium	Yes	0	0				✓	Yes	10	9
Canada	No ^c^	12	12				✓	No ^c^	12	12
Denmark	N/A	N/A	N/A				✓	Yes	9	12
Netherlands	Yes	0	0				✓	No	11	11
UK	Yes	0	9	✓	✓	✓	✓	Yes	12	20
Malaysia	Yes	15	20	✓		✓	✓	Yes	19	14
Philippines	No	9	9				✓	No	13	13
Taiwan	No	17	17				✓	No	0	0
Thailand	No	0	0				✓	Yes	11	8

^a^: Data from survey, ^b^: Data from website of national pharmacovigilance centres, ^c^: The reporting forms for spontaneous reporting between HCP and patient is the same. However, the form for mandatory reporting of serious ADRs is different from spontaneous reporting form. N/A: not available.

**Table 5:** Details of patient reporting form.

Country	Number of Fields ^a^											
	Paper Form						Online Form					
	Total No.	Patient Information	Medicines InforMation	ADR Information	Reporter Information	Additional Information	Total No.	Patient Information	Medicines Information	ADR Information	Reporter Information	Additional Information
Belgium	36	11	10	10	5	-	34	7	12	10	4	1
Canada	39	5	20	5	9	-	45	7	19	6	11	2
Denmark	N/A	N/A	N/A	N/A	N/A	N/A	40	7	9	6	8	10
Netherlands	21	3	6	5	6	1	28	4	10	10	2	2
UK	34	8	10	6	6	4	43	8	10	7	10	8
Malaysia	24	6	6	8	4	-	24	6	6	8	4	-
Philippines	33	8	8	10	7	-	27	5	12	6	2	2
Taiwan	28	6	6	10	6	-	28	6	6	10	6	-
Thailand	31	9	8	7	5	2	18	10	1	3	2	2

^a^: Data from from website of national pharmacovigilance centres, N/A: not available.

**Table 6:** Characteristics of patient paper form.

Country	Mandatory Fields in Patient Paper Form ^a^									
	Patient Information		Medicines Information		Adverse Drug Reaction Information				Reporter Information	
	Age/Birth Date	Gender	Suspected Medicine	Indication	Symptom	Start Date of ADR	Outcome	Severity	Name	Contact
Belgium	No	No	No	No	No	No	No	No	No	No
Canada ^b^	Yes	Yes	Yes	Yes	Yes	Yes	Yes	No	Yes	Yes (Tel. no.)
Denmark	N/A		N/A		N/A				N/A	
Netherlands	No	No	No	No	No	No	No	No	No	No
UK	Yes	No	Yes	No	Yes	No	Yes ^c^	No	Yes	Yes (address)
Malaysia ^d^	Yes	Yes	Yes	Yes	Yes	Yes	Yes	Yes	No	Yes (Tel. no.)
Philippines ^e^	Yes	Yes	Yes	No	Yes	Yes	No	Yes	Yes	Yes (Tel. no.)
Taiwan	Yes	Yes	Yes	No	Yes	Yes	No	Yes	Yes	Yes (Tel no., e-mail, address)
Thailand	No	No	No	No	No	No	No	No	No	No

^a^: Data from website of national pharmacovigilance centres, ^b^: Medicines information: Start date of medicine, Dosage, Route of administration, ^c^: ADR information: Patient can describe feeling about ADR in free-text field, ^d^: Patient information: Patient’s name, Pregnancy, Allergy history, Ethnicity, Medicines information: Concomitant medicine, Start date of medicine, Dose, Frequency, Stop date of medicine, Rechallenge, ADR information: Stop date of ADR, Treatment of ADR, ^e^: Medicines information: Rechallenge, ADR information: Treatment of ADR. N/A: not available.

**Table 7:** Characteristics of patient online form.

Country	Mandatory Fields in Patient Online Form ^a^									
	Patient Information			Medicines Information		Adverse Drug Reaction Information			Reporter Information	
	Name/Initials	Age/Birth Date	Gender	Suspected Medicine	Concomitant Medicine	Symptom	Severity	Outcome	Name	Contact
Belgium	Yes	Yes	Yes	Yes	Yes	Yes	Yes	No	No	No
Canada	Yes	Yes	Yes	Yes	No	Yes	No	No	Yes	Yes (Tel. no., address, e-mail)
Denmark ^b^	Yes	Yes	No	Yes	Yes	Yes	Yes	Yes	Yes	Yes (Tel. no., address)
Netherlands ^c^	Yes	Yes	No	Yes	No	Yes	Yes	Yes	No	Yes (e-mail)
UK ^d^	Yes	No	No	Yes	No	Yes	Yes	Yes	Yes	Yes (address)
Malaysia ^e^	Yes	Yes	Yes	Yes	No	Yes	Yes	Yes	No	Yes (Tel. no., e-mail)
Philippines ^f^	Yes	Yes	Yes	Yes	No	Yes	No	No	Yes	Yes (e-mail)
Taiwan	No	No	No	No	No	No	No	No	No	No
Thailand ^g^	Yes	Yes	Yes	Yes	No	Yes	No	No	No	Yes (Tel. no., address)

^a^: Data from website of national pharmacovigilance centres, ^b^: Patient information: Medical history, Medicines information: Pharmaceutical form, ADR information: Start date of treatment ADR, Stop date of ADR, ^c^: Medicines information: Strength, Dose, start date of medicine, Have you ever received a medicine before? ADR information: Start date of ADR, ^d^: ADR information: How the side effect affected you? Additional information: Did your HCP complete a Yellow card on your behalf? permission to contact reporter and your doctor, permission to send copy report to your HCP, ^e^: Patient information: Pregnancy, Medicines information: Rechallenge, ADR information: Duration, Treatment of ADR, ^f^: ADR information: Start date of ADR, Additional information: Your HCP data, ^g^: ADR information: Start date of ADR.

**Table 8:** Activities regarding adverse drug reaction reporting by patients/consumers.

Country	Provision of Feedback to Reporters ^a^	Methods for Promote ADRs Reporting by Patients ^a,b^			
		Promotion via Media or Social Media	Promotion via HCPs	Promotion via Patient Organizations	Provision of Activities/ Campaign Aimed at General Public
Belgium	Yes	Yes	No	No	Yes
Canada	Yes ^c^	Yes	No	No	No
Denmark	No	Yes	Yes	Yes	No
Netherlands	Yes ^d^	Yes	Yes	Yes	Yes
UK	Yes ^c^	Yes	Yes	Yes	Yes
Malaysia	No	Yes	Yes	No	No
Philippines	No	Yes	No	No	No
Taiwan	No	No	No	No	No
Thailand	No	No	No	No	No

^a^: Data from survey, ^b^: Data from website of national pharmacovigilance centres, ^c^: reporter will receive the link to online ADR report databases received by the pharmacovigilance centre, ^d^: feedback in specific cases (e.g., when doctor advice is need, when the patient asks a question).

## Data Availability

The data presented in this study are available on request from the corresponding author. The data are not publicly available due to the confidentiality policy.
